# Slow miniscrew-assisted palatal expansion: a clinical approach for long-term skeletal stability and enhanced nasal airway function

**DOI:** 10.1007/s11325-026-03669-0

**Published:** 2026-04-14

**Authors:** Rana Kiziltekin Cimen, Choon Bong Lee, Huy Young Kim, Stanley Yung-Chuan Liu, Hwa Sung Chae, Christine Hong

**Affiliations:** 1 Department of Orofacial Sciences, University of California, San Francisco, School of Dentistry, San Francisco, CA USA; 2 Dental Clinic Director, Gounmiso Orthodontic Clinic, Bucheon, South Korea; 3 Department of Family Medicine, Ajou University Hospital, Suwon, South Korea; 4 Department of Oral & Maxillofacial Surgery, Nova Southeastern University, Fort Lauderdale, FL USA; 5 Department of Orthodontics, Institute of Oral Health Science, Ajou University School of Medicine, Suwon, South Korea

**Keywords:** Miniscrew-assisted palatal expander (MAPE), Maxillary transverse hypoplasia (MTH), Cone-beam computed tomography (CBCT), Peak nasal inspiratory flow (PNIF), Nasal airway function

## Abstract

**Purpose:**

To assess whether slow-activation miniscrew-assisted palatal expansion (MAPE) improves skeletal dimensions, nasal airflow and patient-reported symptoms in maxillary transverse hypoplasia (MTH).

**Methods:**

In this prospective cohort, 45 subjects underwent slow-activation MAPE and were stratified as pediatric (< 18y; *n* = 22) and adult (≥ 18y; *n* = 23). Outcomes were collected at baseline (T0), post-expansion (T1), post-orthodontics (T2) and 1-year retention (T3). CBCT quantified zygomatic and nasal widths and internal nasal valve (INV) area/angle and intermolar widths. Peak nasal/oral inspiratory flows (PNIF/POIF) were measured at T0–T3. Patient-reported outcomes included a 23-item binary questionnaire and the Epworth Sleepiness Scale (ESS).

**Results:**

Zygomatic and nasal widths and INV area/angle increased in both cohorts (all *p* < 0.001), with larger INV changes in pediatrics (*p* < 0.05). PNIF increased and remained above baseline at T3 (*p* < 0.001), while morning mouth dryness upon waking and daytime mouth breathing decreased (both *p* < 0.01). Sleepiness outcomes were not uniformly improved. Using Johns ESS categories, from T0 to T3, 67% of pediatric participants shifted to lower-normal and 33% remained higher-normal, whereas all adults remained higher-normal. Using a two-group threshold informed by Korean validation (ESS ≥ 8 vs. < 8), 35% were ≥ 8 at baseline; at T3, all pediatric participants and 25% of adults who were ≥ 8 shifted to < 8, while 75% remained ≥ 8.

**Conclusions:**

Slow-activation MAPE was associated with skeletal and nasal complex widening, INV enlargement, and sustained PNIF improvement, but daytime sleepiness improved inconsistently, particularly in adults. Without polysomnography, findings reflect changes in upper-airway physiology and symptoms rather than definitive changes in sleep-disordered breathing severity.

## Introduction

Maxillary growth deficiency can cause structural and functional problems, including malocclusion, aesthetic concerns, and airway compromise [1, 2]. A common presentation is maxillary transverse hypoplasia (MTH), often with a high-arched palate and narrow nasal cavity with associated elevated nasal airway resistance [1, 3]. Restricted nasal airflow promotes mouth-breathing during daytime and sleep, which can disrupt diaphragmatic mechanics and respiratory-muscle activity [2, 3].

Treatment planning prioritizes the degree of MTH using transverse analyses (e.g., Yonsei), rather than dental crowding alone [1, 4]. When the discrepancy is primarily dentoalveolar or has a minor skeletal component, tooth-borne expansion (slow or rapid maxillary expansion(RME) ~ 1.4 mm/week) can suffice [1, 5, 6]. When a predominantly orthopedic correction is required, bone-borne techniques, such as maxillary skeletal expander (MSE), miniscrew-assisted rapid palatal expansion (MARPE) or miniscrew-assisted palatal expansion (MAPE) with slow protocol, are preferred and have shown reliable outcomes [4–7]. These approaches deliver forces directly to bone, achieving greater skeletal expansion while minimizing dental side effects [5]. 

Rapid bone-borne protocols (~ 2.8 mm/week) shorten treatment time but may overload circum-maxillary sutures, increasing asymmetric expansion, soft-tissue irritation and midline diastema, especially with high skeletal resistance [4, 6, 8, 9]. Slow protocols (~ 0.5–1 mm per week) achieve the required transverse correction over a longer period while minimizing dental side effects and allowing the circum-maxillary sutures and surrounding tissues to adapt gradually [5, 10]. These distinctions highlight the clinical advantages of a slow, bone-borne expansion protocol in substantial MTH. Bone-borne expansion can increase nasal cavity volume and upper-airway dimensions [2, 9, 11] and may promote a more anterior–superior tongue posture; these changes have been linked to improved nasal airway physiology [2, 9, 10, 12]. 

Because MTH narrows the nasal cavity and increases resistance [3, 8], we assessed the skeletal and functional effects of slow-activation MAPE [8]. Participants were stratified a priori into pediatric (< 18 y) and adult (≥ 18 y) cohorts to limit growth confounding [8, 13]. To evaluate functional impact, we measured peak nasal inspiratory flow (PNIF) [2, 14, 15] as an index of nasal patency and peak oral inspiratory flow (POIF) [16] as an index of oral airflow and combined these with patient-reported outcomes (Sleep-Related Binary Questionnaire and Epworth Sleepiness Scale) [12, 14].

Using this multimodal approach, we investigated whether slow-activation MAPE can correct skeletal MTH, enlarge the nasal airway, improve inspiratory flow patterns and translate into patient-reported outcomes.

## Materials and methods

### Subjects

This study complied with institutional/national ethics and the requirement for informed consent was waived by the IRB because the study involved minimal risk to subjects and there was no reasonable expectation that participants would refuse consent (IRB approvals: AJO-2019-389; AJOUIRB-MDB-2022-566). Forty-five subjects met inclusion/exclusion criteria (pediatric *n* = 22; adult *n* = 23).

A miniscrew-assisted palatal expansion (MAPE) device with four miniscrews and a central jackscrew was used; screw dimensions were selected to palatal bone (diameter 1.8–2.0 mm; length 9–13 mm). Measurements were obtained at four time points: baseline (T0), post-expansion (T1), post-orthodontics (T2), and 1-year retention (T3). CBCT scans were acquired at T0, T1, and T2. PNIF and POIF were recorded at T0–T3. The 23-item Sleep-Related Binary Questionnaire and the Epworth Sleepiness Scale (ESS) were administered at T0 and T3.

#### Inclusion

permanent dentition and skeletal MTH based on the Yonsei Transverse Index (YTI).

#### Exclusion

impacted teeth, history of oral trauma, systemic disease affecting tooth movement (e.g., cleidocranial dysplasia, cleft palate) and prior extractions.

### CBCT measurements

**Fig. 1:**
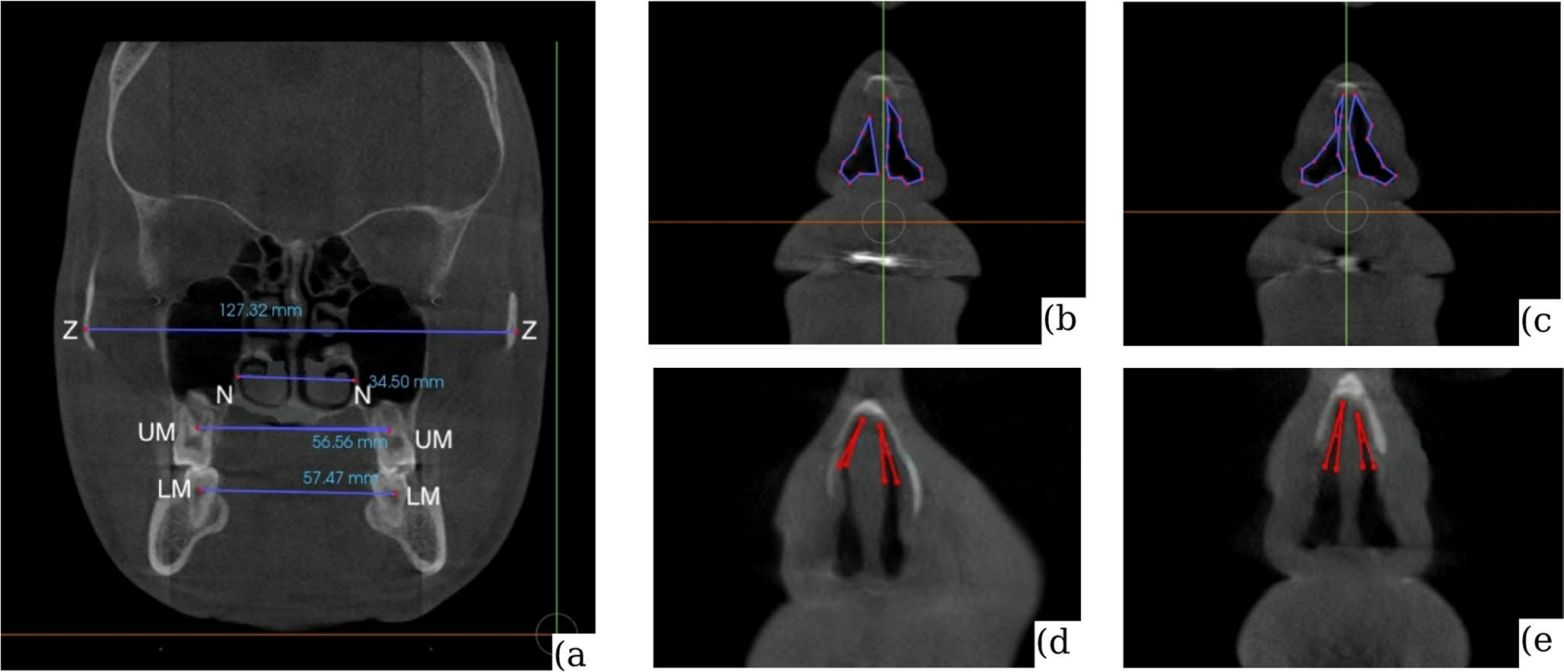
Landmarks and measurements on coronal CBCT scans: (**a**) reference landmarks: Z, most lateral point of zygomatic arch; N, most lateral point of nasal cavity; UM, furcation of upper first molar; LM, furcation of lower first molar. (**b**) INV cross-sectional area, preoperative. (**c**) INV area, postoperative. (**d**) INV angle, preoperative. (**e**) INV angle, postoperative

CBCT (InVivoDental^®^, v5.1, Anatomage) was used to measure nasal and zygomatic widths and upper/lower first-molar distances [4, 9, 13]. The internal nasal valve (INV) was quantified on standardized coronal reconstructions as a CBCT surrogate of the clinical INV; right/left area and angle were measured and averaged [9, 12, 13]. For nasal and zygomatic widths, the outermost bony points were identified [4, 9, 13] (Fig. 1).

Transverse discrepancy was assessed with YTI, which indexes the distance between the centers of resistance of the maxillary and mandibular first molars, reflecting basal arch width rather than cusp-tip distance [4, 17]. Because the mandibular base is normally wider at the CR level, YTI guided target expansion magnitude [1, 4, 17]. 

### Inspiratory flow measurements

PNIF provides a rapid, objective measure of nasal airflow; normative values exist for adults and children [14–16, 18]. POIF quantifies maximal inspiratory flow through the mouth and serves as a proxy for oral compensatory breathing when nasal resistance is elevated [14, 15]. Together, PNIF and POIF characterize nasal patency and mouth-breathing compensation relevant to SRBD [3, 14]. 

A handheld inspiratory flow meter, In-Check Nasal (Clement Clarke International Ltd., UK), (range 0–340 L/min) recorded PNIF and POIF. For PNIF, subjects were seated with neutral head position, a soft nasal mask seal, closed lips, and a rapid maximal nasal inspiration [14–16]. For POIF, inspiration occurred via the device mouthpiece with nares occluded [14, 15]. Each effort began after a relaxed exhalation; the device output peak flow (L/min). Each measure was obtained in triplicate with brief rests; the best reproducible value within a preset variability threshold was analyzed [14, 16]. Visits were scheduled at similar times when feasible; acute upper-respiratory infection/allergic flare triggered deferral. Device hygiene and calibration followed manufacturer instructions.

### Expander activation protocol and orthodontic treatment

**Scheme 1:**
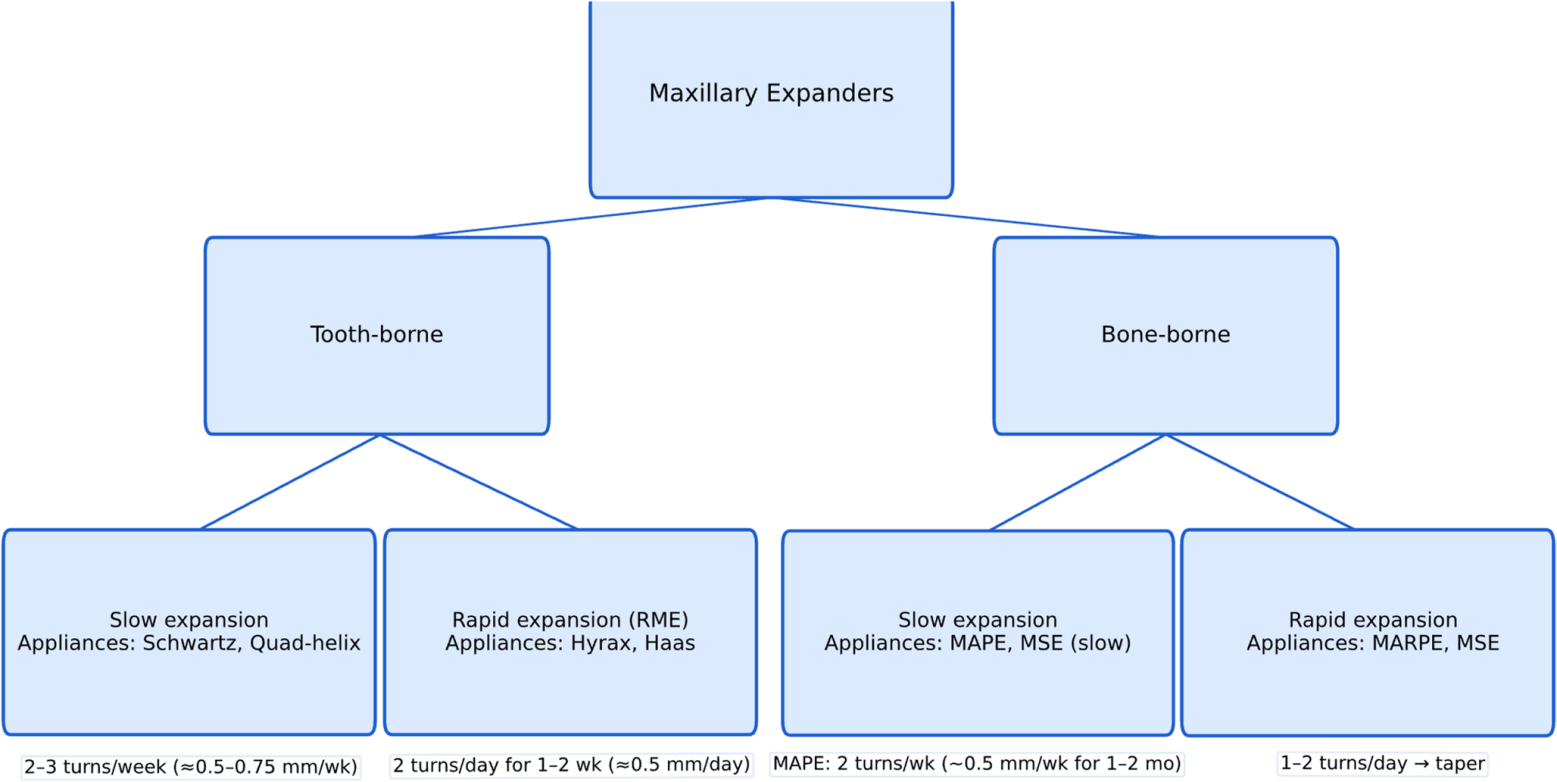
Taxonomy of maxillary expanders: tooth-borne vs. bone-borne, each subdivided into *slow* and *rapid* activation. Boxes list representative appliances (e.g., tooth-borne slow: *Schwartz*,* quad-helix*; tooth-borne rapid/RME: *Hyrax*,* Haas*; bone-borne slow: *MAPE/MSE-slow*; bone-borne rapid: *MARPE/MSE*). Protocol annotations are shown adjacent to each branch

Expansion began immediately after MAPE placement under specialist supervision. The axial screw was turned twice weekly (0.5 mm/week) for an initial period of 1–2 months, followed by twice-monthly (0.5 mm/visit) until the YTI-defined expansion target was approached [17]. The initial activation was designed to confirm stability of the bone-borne anchorage screws and to monitor for palatal soft-tissue irritation during clinician-performed activation. In most cases, this verification period was completed within approximately 1 month; in selected cases requiring additional monitoring, it was extended to up to 2 months. Planned expansion aimed to approximate YTI normalization (approximately YTI = 0); however, the final expansion endpoint was guided by clinical stability and occlusal relationships rather than achievement of an exact numeric YTI value, and no intentional overcorrection was applied. Notably, “twice monthly activation” refers to two consecutive activations performed during a single monthly visit, rather than activations spaced approximately 15 days apart. This slow, bone-borne protocol was applied uniformly to pediatric and adult subjects. Comprehensive fixed-appliance orthodontics followed to finalize occlusion (Scheme 1).

### Statistical analysis

Analyses were performed in SPSS 26.0. An a priori power analysis (G*Power 3.1.9.4) for repeated-measures ANOVA with four levels (f = 0.25, α = 0.05, 1–β = 0.90) indicated *N* = 40; the observed PNIF time effect approximated f ≈ 0.50. Normality was screened via skewness/kurtosis (− 2 to + 2). For each cohort (< 18 y; ≥18 y), variables at T0–T3 are reported as mean ± SD. Intra-rater reliability was assessed using intraclass correlation coefficients (two-way mixed-effects model, absolute agreement; ICC (3,1) and ICC (3,2)) with 95% confidence intervals from repeated measurements by the same examiner.

Within-subject changes over time (T0–T3) were tested using repeated-measures ANOVA. When Mauchly’s test indicated violation of sphericity (*p* < 0.05), Greenhouse–Geisser corrections were applied. Significant omnibus effects were followed by Bonferroni-adjusted pairwise comparisons and polynomial contrasts (linear, quadratic) to characterize trends. Partial η² is reported as the primary effect size. Pre/post subjective measures were compared with paired t-tests. ESS was interpreted primarily using Murray Johns’s categorical ranges and T0–T3 category transitions (improved/unchanged/worsened) were summarized for both cohorts. As a secondary interpretation, we analyzed scores using a two-group cutoff (ESS ≥ 8 vs. < 8), informed by the Korean validation study’s reported control and patient reference values. Two-tailed *p* < 0.05 was considered significant.

## Results

Forty-five subjects underwent slow-activation MAPE and had pre- and post- treatment data available for analysis (9 males [20%], 36 females [80%]). Baseline age was 20.1 ± 6.9 years (range, 11–37). Participants were stratified a priori into pediatric (< 18 years; *n* = 22) and adult (≥ 18 years; *n* = 23) cohorts to minimize growth-related confounding. Intra-rater reliability was assessed using ICC (3,2) (*n* = 45). Linear measures showed excellent reliability (ICC(3,2): 0.808–0.993; 95% CI lower bounds 0.65–0.99, upper bounds 0.89–1.00) with mean error differences (M2 − M1) of − 0.558 to 0.856 mm. Area measures also demonstrated excellent reliability (ICC(3,2): 0.972–0.980; 95% CI lower bounds 0.95–0.96, upper bounds 0.98–0.99) with mean error differences of 0.133 to 1.146 mm². Angle measures showed lower reliability (ICC (3,2): 0.296–0.712; 95% CI lower bounds − 0.28–0.48, upper bounds 0.61–0.84) with mean error differences of − 0.158 to 0.127°. Demographic characteristics are summarized (Table 1).

**Table 1:** Demographic distribution

Cohort	*N* ^1^	Age, mean ± SD^2^ (range)	Sex, *N* (%)
Total	45	20.1 ± 6.9 (11.1–37.8)	Female 36 (80%), Male 9 (20%)
Pediatric (< 18 y)	22	14.9 ± 2.2 (11.1–17.9)	Female 18 (82%), Male 4 (18%)
Adult (≥ 18 y)	23	25.0 ± 6.3 (18.3–37.8)	Female 18 (78%), Male 5 (22%)

^1^*N* Number, ^2^*SD* Standard Deviation

### Dental measurements

**Fig. 2:**
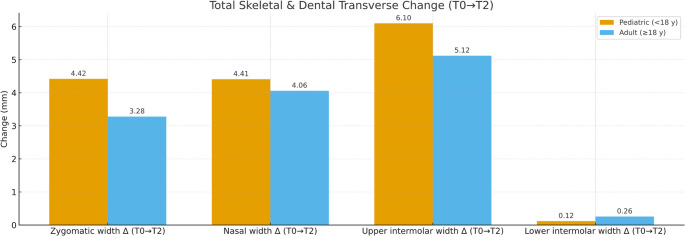
Total transverse change (T0–T2) after slow-activation MAPE. Bars show mean Δ (mm) for zygomatic width, nasal width, upper intermolar, and lower intermolar by cohort (pediatric < 18 y; adult ≥ 18 y). Zygomatic, nasal, and upper intermolar increased significantly within cohorts (all *p* < 0.001); lower intermolar showed no time effect. Between-cohort differences in Δ(T2–T0) were not significant

This analysis evaluated whether slow-activation MAPE alters maxillary and mandibular intermolar widths, clarifying whether changes are primarily orthopedic rather than dentoalveolar (Tables 2 and 3; Fig. 2).

**Table 2:** Independent-samples t-test for between-group changes

Independents	Group	*N*	Mean	SD	t	*p*
Change POIF (T3^3^-T0^1^)	Pediatric	22	33.1739	49.9001	1.219	0.229
	Adult	23	11.3043	63.05152		
Change PNIF (T3-T0)	Pediatric	22	58.1013	28.35928	1.751	0.087
	Adult	23	41.087	34.50812		
Change Zygomatic Width (T2^2^-T0)	Pediatric	22	4.4191	4.15895	1.100	0.277
	Adult	23	3.2774	2.73807		
Change Nasal Width (T2-T0)	Pediatric	22	4.4087	1.95529	0.350	0.728
	Adult	23	4.0583	4.38385		
Change The Upper Intermolar Width (T2-T0)	Pediatric	22	6.1	2.14191	1.374	0.177
	Adult	23	5.1148	2.69182		
Change The Lower Intermolar Width (T2-T0)	Pediatric	22	0.1187	0.96419	− 0.341	0.734
	Adult	23	0.2565	1.67853		
Change INV Angle (T2-T0)	Pediatric	22	2.9957	1.33527	2.028	0.049*
	Adult	23	2.1522	1.48213		
Change INV Area (T2-T0)	Pediatric	22	27.4707	14.27777	2.310	0.026*
	Adult	23	22.3437	11.60798		

^1^T0: baseline, ^2^T2: post-orthodontics, ^3^T3: 1-year retention, * *p* < 0.05, statistically significant

**Table 3:** Effect sizes (partial η²) of time-dependent changes, by group

Dependent	Group	η²*p*	Effect-Size*
PNIF	Pediatric	0.86	Very Large
PNIF	Adult	0.81	Very Large
POIF	Pediatric	0.58	Large
POIF	Adult	0.49	Large
Zygomatic Width	Pediatric	0.65	Large
Zygomatic Width	Adult	0.69	Large
Nasal Width	Pediatric	0.80	Very Large
Nasal Width	Adult	0.76	Very Large
Upper Molars Width	Pediatric	0.93	Very Large
Upper Molars Width	Adult	0.89	Very Large
Lower Molars Width	Pediatric	-	Not meaningful
Lower Molars Width	Adult	-	Not meaningful
INV Angle	Pediatric	0.84	Very Large
INV Angle	Adult	0.80	Very Large
INV Area	Pediatric	0.81	Very Large
INV Area	Adult	0.78	Very Large

*Interpretations follow conventional thresholds for partial η² (≈ 0.01 = small, 0.06 = medium, 0.14 = large); values above ≈ 0.80 are often described as “very large.”

Maxillary intermolar width (mm) increased significantly in both cohorts with very large effects (pediatrics η²*p* = 0.933; adults η²*p* = 0.887). Pediatrics showed a larger early gain: +5.59 mm T0→T1 (*p* < 0.001) and + 0.51 mm T1→T2 (*p* = 0.817), totaling **+** 6.10 mm T0→T2 (*p* < 0.001). Adults were similar: +4.04 mm T0→T1 (*p* < 0.001) and + 1.08 mm T1→T2 (*p* = 0.116), totaling + 5.12 mm T0→T2 (*p* < 0.001). No between-group differences were significant.

Mandibular intermolar width (mm) showed no significant change across time in either cohort. Between-group differences in the T2–T0 change were not significant for upper and lower intermolar width (Tables 2 and 3; Fig. 2).

### Skeletal measurements

Zygomatic and nasal width tests whether slow-activation MAPE produces skeletal changes extending beyond the midpalatal suture into the zygomatic/nasal region, as documented in CBCT and modeling studies of bone-borne expansion [4] (Tables 2 and 3; Fig. 2).

Zygomatic width increased significantly in both cohorts with large effects (pediatrics η²*p* = 0.587; adults η²*p* = 0.687). Pediatrics: +1.74 mm T0→T1 (*p* = 0.036), + 2.68 mm T1→T2 (*p* < 0.001), total + 4.42 mm T0→T2 (*p* < 0.001). Adults: +1.51 mm T0→T1 (*p* < 0.001), + 1.77 mm T1→T2 (*p* = 0.001), total + 3.28 mm (*p* < 0.001). Between-group Δ(T2–T0) did not differ.

Nasal width increased with very large effects (pediatrics η²*p* = 0.917; adults η²*p* = 0.806). Pediatrics showed a larger early gain: +2.90 mm T0→T1 (*p* < 0.001) and + 1.51 mm T1→T2 (*p* = 0.001), total + 4.41 mm (*p* < 0.001). Adults: +2.22 mm T0→T1 (*p* < 0.001), non-significant T1→T2 (*p* = 0.105), total + 4.06 mm (*p* = 0.001). No between-group differences were significant.

### Internal nasal valve area and angle

**Fig. 3:**
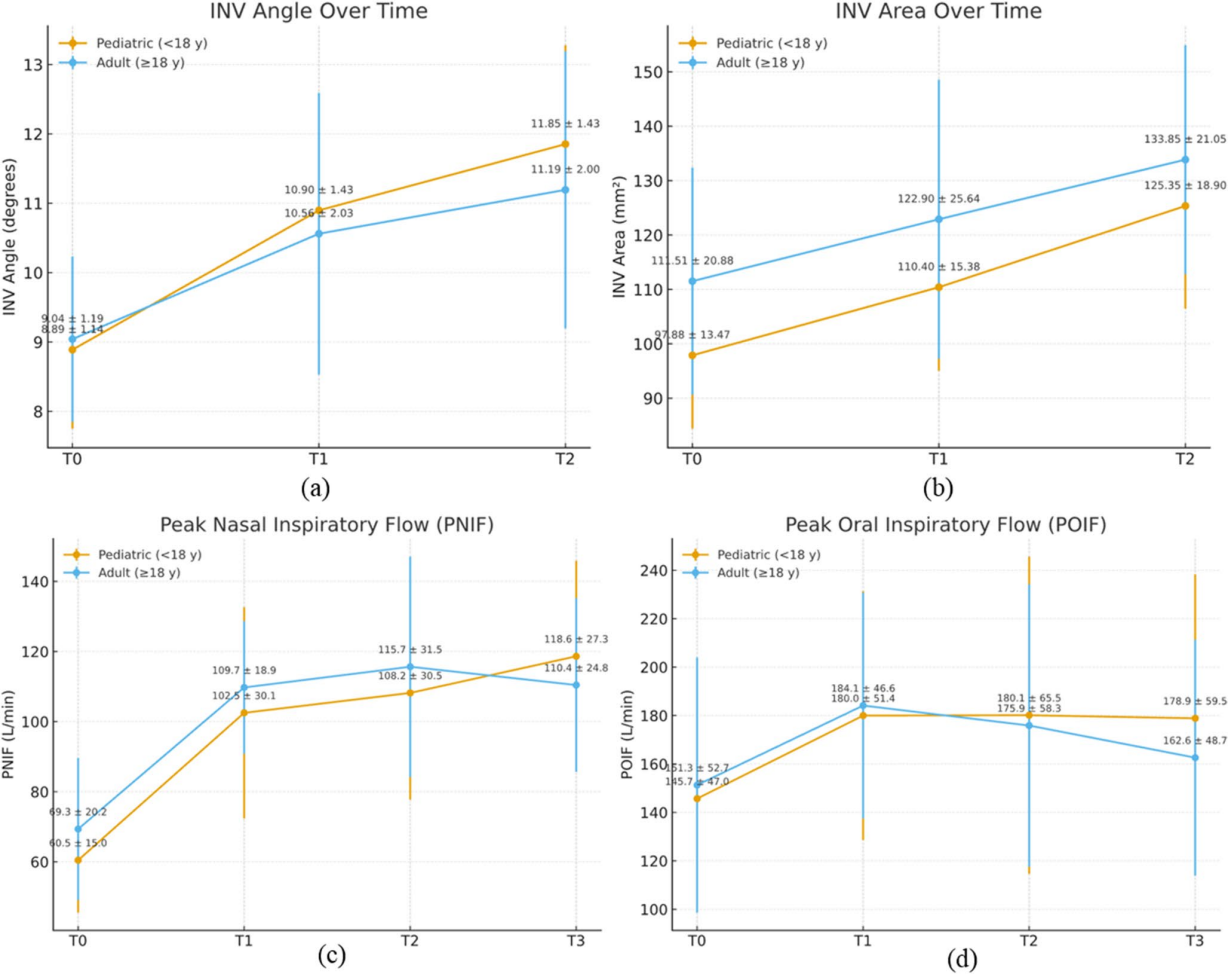
(**a**–**b**) Internal nasal valve (INV) angle (°) and area (mm²) at T0, T1, T2 and (**c**-**d**) peak nasal (PNIF) and oral (POIF) inspiratory flow (L/min) at T0–T3, by cohort (pediatric < 18 y; adult ≥ 18 y). Points show mean ± SD. INV angle and area increased in both cohorts to T2 (all *p* < 0.001), with larger pediatric Δ(T2–T0). PNIF rose from baseline and remained elevated through T3 in both cohorts (all *p* < 0.001). POIF increased during expansion, then stabilized (pediatric) or trended toward baseline (adult)

INV angle (°) increased significantly in both cohorts, with very large effects (pediatrics η²*p* = 0.840; adults η²*p* = 0.703). In pediatrics, results showed linear and quadratic components (larger early gain): 2.07° (T0→T1, *p* < 0.001), 0.93° (T1→T2, *p* = 0.001), total 3.00° (T0→T2, *p* < 0.001). In adults, a similar pattern was observed: 1.52° (T0→T1, *p* < 0.001), 0.63° (T1→T2, *p* < 0.001), total 2.15° (T0→T2, *p* < 0.001). Between-group Δ(T2–T0) differed (*p* = 0.049), with a greater increase in pediatrics (Tables 2 and 3; Fig. 3).

INV area (mm²) increased significantly in both cohorts, with very large effects (pediatrics η²*p* = 0.785; adults η²*p* = 0.809). Pediatrics: linear pattern; mean increased by 12.52 mm² from T0 to T1 (*p* < 0.001) and 14.95 mm² from T1 to T2 (*p* < 0.001), totaling 27.47 mm² from T0 to T2 (*p* < 0.001). Adults: also, linear; 11.39 mm² from T0 to T1 (*p* = 0.001) and 10.96 mm² from T1 to T2 (*p* < 0.001), totaling 22.34 mm² from T0 to T2 (*p* < 0.001). Between-group Δ(T2–T0) differed (*p* = 0.026), with a greater increase in pediatrics.

### Peak nasal and oral inspiratory flows

Peak flows were evaluated to assess whether slow-activation MAPE produces functional respiratory gains, particularly at the anterior–inferior nasal floor/internal nasal valve, rather than merely the skeletal or dental changes reported previously [10, 17, 19–21] (Tables 2 and 3; Fig. 3).

PNIF (L/min) increased markedly over time in both cohorts, with very large effects (pediatrics η²*p* = 0.861; adults η²*p* = 0.805). In pediatrics, means rose from 60.5 at T0 to 118.6 at T3; trend analysis showed linear, quadratic, and cubic components; indicating a large early rise (T0→T1) with smaller increments thereafter. Bonferroni tests found T0 < T1/T2/T3 (all *p* < 0.001), while T1, T2, and T3 did not differ. In adults, means increased from 69.35 at T0 to 110.43 at T3; linear and quadratic trends were significant, the cubic was not. Pairwise results mirrored pediatrics: T0 < T1/T2/T3 (all *p* < 0.001), with no differences among follow-ups.

POIF (L/min) changed significantly over time in both cohorts, with large effects (pediatrics η²*p* = 0.582; adults η²*p* = 0.486). In pediatrics, means rose from 145.7 at T0 to 178.9 at T3; repeated-measures ANOVA with Greenhouse–Geisser correction was significant (F (2.325, 51.156) = 4.975, *p* = 0.008). Trend analysis showed linear and quadratic components and Bonferroni tests confirmed that T0 differed from T1, T2, and T3 (all *p* ≤ 0.036), whereas T1, T2, and T3 did not differ. In adults, means increased from 151.30 (T0) to a peak at 184.13 (T1), then declined toward 162.61 (T3); the Greenhouse–Geisser ANOVA was significant (F (2.050, 45.089) = 3.468, *p* = 0.039). Trend analysis indicated a quadratic pattern without a linear component, and pairwise comparisons identified T0 vs. T1 as the only significant difference (*p* = 0.004), with no other follow-up contrasts reaching significance.

### Binary questionnaire and Epworth sleepiness analysis

**Fig. 4:**
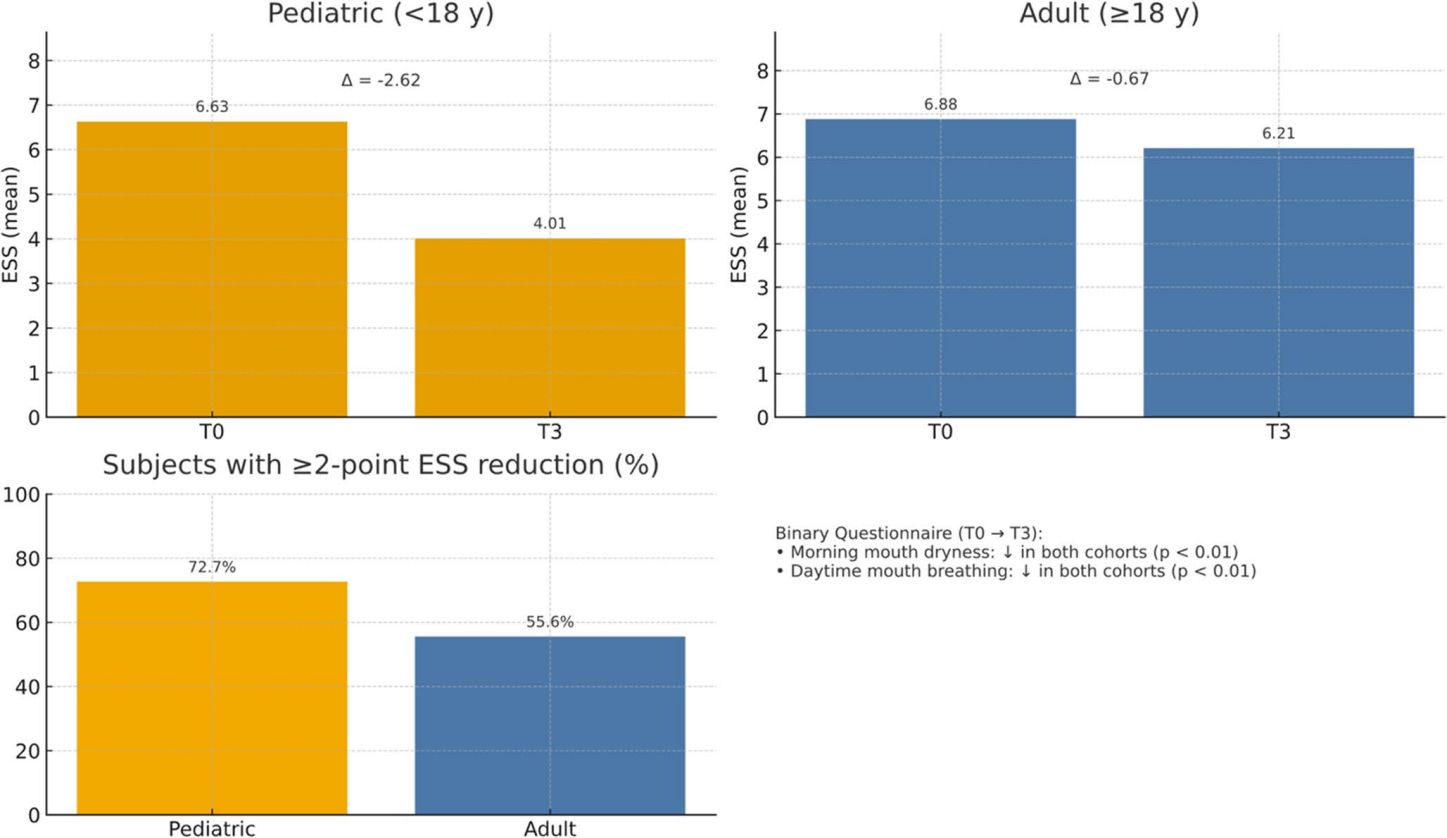
Epworth sleepiness scale (ESS) and questionnaire outcomes after slow-activation MAPE. Pediatric and adult mean ESS at baseline (T0) and 1-year retention (T3); labels show ΔT3–T0. Percentage of subjects achieving a ≥ 2-point ESS reduction. Binary questionnaire summary reports of morning mouth dryness and daytime mouth breathing decreased in both cohorts (*p* < 0.01)

Binary questionnaire outcomes (23 items) were obtained at baseline (T0) and at 1-year retention (T3). In both cohorts, the proportion of participants reporting morning mouth dryness upon waking and daytime mouth breathing decreased significantly after treatment (*p* < 0.01), consistent with improved nasal breathing following slow-activation MAPE (Fig. 4).

Using Murray Johns’s ESS categories (0–5 lower normal, 6–10 higher normal, 11–12 mild excessive daytime sleepiness, 13–15 moderate excessive daytime sleepiness, 16–24 severe excessive daytime sleepiness), 65% of participants (45% pediatric, 20% adult) were classified as higher-normal (6–10) and 35% as lower-normal (0–5) at baseline. At T3, 67% of pediatrics shifted to the lower-normal category and 33% remained higher-normal, whereas all adults remained higher-normal. Using the two-group cutoff informed by Korean ESS reference values (ESS ≥ 8 vs. < 8), 35% of participants (15% pediatric, 20% adult) were classified as high ESS at baseline. At T3, all pediatrics were classified as low ESS; among adults with high ESS at baseline, 25% shifted to low ESS and 75% remained high.

In the pediatric cohort, 72.7% showed a reduction in ESS, 9.1% were unchanged, and 18.2% increased; the mean ESS decreased from 6.63 at T0 to 4.01 at T3 (Δ = −2.62).

In the adult cohort, 55.6% improved, 22.2% were unchanged, and 22.2% increased; the mean ESS decreased from 6.88 at T0 to 6.21 at T3 (Δ = −0.67).

Overall, patient-reported outcomes indicate improvements in nasal obstruction symptoms and daytime sleepiness, with larger effects in the pediatric cohort.

## Discussion

Slow-activation MAPE addressed maxillary transverse deficiency with skeletal expansion, nasal airway enlargement and functional breathing improvements while limiting mandibular dental change; the combined skeletal, dentoalveolar, INV geometry, peak inspiratory flows and patient-reported outcomes support an airway-focused benefit.

### Dental evaluation

MAPE targets skeletal MTH while minimizing dentoalveolar effects. Clinically, MTH typically presents as a narrow, high-arched maxilla with posterior crossbite; dental crowding may coexist but is not diagnostic [1, 22]. Maxillary constriction narrows the nasal cavity and elevates nasal airflow resistance and has been linked to breathing disturbances [3, 23]. Habitual mouth breathing is associated with altered orofacial muscle balance and a lower tongue posture and corresponds to reduced nasal inspiratory flow [2, 14, 23]. 

Compared with tooth-borne rapid expansion (risk of buccal tipping/diastema), bone-borne slow activation distributes load across mid-palatal and circum-maxillary sutures, favoring orthopedic change with fewer dental effects [21]. Here, maxillary intermolar width increased early then stabilized (T0→T1 ≫ T1→T2), while mandibular width remained largely unchanged, consistent with prior reports [5, 21]. Expansion may enlarge the palatal vault, facilitating a more anterior–superior tongue posture and reduced mouth breathing, consistent with observed INV enlargement and sustained PNIF gains [2, 10, 22]. Improvements were evident in both age groups and align with evidence that MARPE benefits extend across ages [24]. 

### Skeletal evaluation

Skeletal change magnitude underpins long-term stability and airway benefit [5, 21, 24]. We observed substantial increases in zygomatic and nasal widths: zygomatic ~ 4.4 mm in pediatrics and ~ 3.3 mm in adults (T0→T2), nasal ~ 4.4 mm in pediatrics and ~ 4.1 mm in adults (about 13–14% overall, T0→T2). Pediatric nasal gains continued T1→T2, whereas adults showed an early gain with subsequent stabilization, patterns consistent with gradual force adaptation and in pediatrics, residual growth potential. Prior rapid protocol reports smaller midfacial changes [4, 6, 25], suggesting that a slow approach may facilitate consolidation of skeletal gains through the finishing phase.

The pediatric group demonstrated greater skeletal increases than the adult group; however, these findings may in part reflect residual craniofacial growth rather than a true treatment effect, and the clinical significance of this difference remains uncertain. Moreover, adolescence may represent a developmental period characterized by heightened skeletal responsiveness and enhanced soft-tissue adaptability, which could further confound direct comparisons with adult outcomes.

Because the INV is the main resistor of nasal airflow, even modest geometric changes can meaningfully reduce inspiratory resistance [19, 20, 23]. Consistent with CBCT/rhinometry literature showing broader nasal dimensions after bone-borne expansion [8, 11, 13, 19, 26], we found significant INV enlargement: area increased by ~ 27.5 mm² (pediatrics) and ~ 22.3 mm² (adults) and angle + 3.00° and + 2.15°, comparable to prior reports [19, 20]. Greater pediatric change (*p* < 0.05) suggests less mature lateral nasal walls and sutures can be more responsive to orthopedic loading [25]. 

### Functional evaluation

PNIF is a sensitive, objective index of nasal patency [15, 16]. Consistent with evidence that expansion reduces nasal resistance and increases nasal volume [10, 26]. PNIF rose substantially and durably relative to baseline: pediatrics 67% at T1, 76.6% at T2, and 93.6% at T3; adults 58.2% at T1, 66.8% at T2, and 59.2% at T3.

POIF increased during active expansion but not thereafter. In pediatrics it stayed modestly above baseline (22.9% T1; 23.0% T2; 21.8% T3), whereas in adults it peaked early (21.7% T1) and then declined toward baseline (16.2% T2; 7.5% T3).

Pediatric gains were larger likely because children have more compliant midfacial/valve tissues, so the similar bone-borne expansion produces greater INV widening [19, 26]. They also start with lower baseline PNIF, so equal absolute increases yield bigger percent gains [22, 27, 28]. Finally, earlier neuromuscular adaptation, less mouth breathing and improved tongue posture, can amplify nasal flow improvements [2]. Adults still showed clear skeletal enlargement and PNIF improvement, consistent with MARPE studies showing increased nasal volume and patency; [4, 9, 25]their PNIF tended to peak earlier and plateau, plausibly reflecting stiffer lateral nasal walls/valve tissues, yet the changes remain clinically significant for symptom relief.

Together, sustained PNIF improvement without a persistent POIF rise indicates a shift toward nasal over oral breathing during follow-up [13, 14, 29], a pattern consistent with prior expansion literature showing decreased nasal resistance and improved nasal patency [13, 14, 29]. 

### Sleepiness evaluation

Although polysomnography was not performed, daytime sleepiness was assessed with the Epworth Sleepiness Scale (ESS) and interpreted using the scale’s established categorical ranges. Using Murray Johns’s ESS categories [29], participants were within the normal range at baseline (65% [45% pediatrics, 20% adults] higher-normal [6–9] and 35% lower-normal [0–5]). From T0 to T3, pediatric participants shifted toward lower-normal sleepiness (67% lower-normal; 33% higher-normal), whereas adults showed no categorical change (all remained higher-normal). (Figure 4).

As a secondary analysis, we also summarized ESS scores using a two-group, Korean-referenced threshold (ESS ≥ 8 vs. < 8), informed by the Korean validation study’s reference values, in which healthy controls had lower mean KESS scores than sleep-related breathing disorder patients (5.07 ± 2.93 vs. 8.21 ± 4.23) [30]. This secondary summary showed a consistent pattern, with improvement primarily observed in the pediatric cohort.

Dr. Murray Johns subsequently developed a pediatric/adolescent adaptation of the ESS (ESS-CHAD) with modifications to adult-oriented contexts [31]. At the time of study initiation, the standard ESS was applied uniformly to maintain consistency between pediatric and adult cohorts; however, we acknowledge that ESS-CHAD is the preferred validated instrument for children/adolescents.

Binary questionnaire items related to oral breathing also improved. The prevalence of morning mouth dryness upon waking and daytime mouth breathing decreased significantly from T0 to T3 (both *p* < 0.01; Fig. 4). These findings complement our objective airway function data, as sustained increases in PNIF and INV area provide physiologic support for improved nasal breathing. Similar links between enhanced nasal patency and reduced sleep-related symptoms have been described by Yoon et al. [19, 20], who noted parallel enhancements in nasal patency and sleep-related symptoms after maxillary expansion [12, 19, 20, 23, 32]. 

Overall, slow bone-borne expansion may support symptom improvement, particularly in growing patients, by enhancing nasal patency without increasing reliance on oral airflow. Given the absence of polysomnography and the use of the standard ESS interpretive frameworks in younger participants, these results should be interpreted as symptom and physiology associations rather than proof of OSA modification. Future studies should incorporate objective sleep testing and pediatric-validated instruments, alongside standardized nasal symptom measures.

### Strengths, limitations, and future directions

Strengths of this study include extended longitudinal follow-up (T0–T3), inclusion of both pediatric and adult cohorts, and multimodal outcome assessment integrating CBCT imaging, inspiratory airflow measurements, and patient-reported outcomes. Key limitations include the absence of polysomnography and three-dimensional volumetric airway analyses, lack of skeletal maturation assessment in the pediatric cohort to account for growth-related changes, a predominance of female participants, and the absence of an untreated or alternative-treatment control group. Daytime sleepiness was assessed using the standard ESS rather than ESS-CHAD; therefore, sleepiness findings in the pediatric cohort should be interpreted cautiously. Future prospective, controlled studies with larger and more demographically diverse cohorts, and objective sleep testing are warranted to clarify the clinical significance of MAPE.

## Conclusions

In patients with maxillary transverse hypoplasia, slow-activation MAPE was associated with measurable skeletal, dental and airway-related changes. Increases were observed in nasal width (+ 4.41 mm in pediatrics and + 4.06 mm in adults) and zygomatic width (+ 4.42 mm in pediatrics and + 3.28 mm in adults). These changes were accompanied by widening of the internal nasal valve (INV) area (+ 27.47 mm² in pediatrics and + 22.34 mm² in adults) and an increase in INV angle (+ 3.00° in pediatrics and + 2.15° in adults), along with sustained improvements in peak nasal inspiratory flow (PNIF) (+ 58.10 L/min, + 96.0% in pediatrics; +41.09 L/min, + 59.2% in adults). Mandibular intermolar width remained stable. Patient-reported outcomes, including morning mouth dryness upon waking and mouth-breathing symptoms, also improved.

However, sleepiness outcomes were not uniformly improved. From T0 to T3, 67% of pediatric participants shifted to the lower-normal ESS category and 33% remained higher-normal, whereas all adults remained higher-normal. Using the Korean-referenced two-group threshold (ESS ≥ 8 vs. < 8), 35% of participants were ≥ 8 at baseline. At T3, all pediatric participants and 25% of adults who were ≥ 8 at baseline shifted to < 8, while 75% of those adults remained ≥ 8.

Overall, slow-activation bone-borne expansion appears to enhance nasal patency and reduce mouth-breathing symptoms, but functional improvement in daytime sleepiness was more limited, particularly in adults. Because polysomnography was not performed, these findings should be interpreted as improvements in symptoms and upper-airway physiology rather than definitive changes in obstructive sleep apnea severity.

## Data Availability

De-identified data underlying the results (aggregated CBCT measurements, PNIF/POIF values, and analysis outputs) are available from the corresponding author upon reasonable request and subject to data-use and privacy restrictions. Raw CBCT image files are not publicly shareable to protect participant privacy.
